# “Fanged to Peril”: Interesting Snake Bite Case Series

**DOI:** 10.7759/cureus.53319

**Published:** 2024-01-31

**Authors:** Vivek Mahadevaiah, Kalpana Ravikumar, Harismitha Manjunath

**Affiliations:** 1 Department of Emergency Medicine, Vydehi Institute of Medical Sciences and Research Center, Bangalore, IND

**Keywords:** anti-snake venom, coagulopathy, broken neck syndrome, vicc, snake bite

## Abstract

Snakebite is an acute life-threatening medical emergency and is included among the neglected tropical diseases in India. The incidence of snakebite mortality is particularly high in Southeast Asia and India has the highest number of cases of snakebites with a mortality of 35,000 to 50,000 cases per year according to the World Health Organization (WHO) direct estimation. There are three families of venomous snakes in Southeast Asia which mainly include Elapidae, Viperidae, and Colubridae. Snakebite victims show varied clinical presentations ranging from local signs to systemic signs of envenomation. WHO has detailed five syndromes based on various clinical presentations and the most probable family of snakes responsible for that syndrome. There are several cases reported on the initial clinical presentations in the literature; however, case series which include the presentation to ER, the hospital course, and management of complications have not been published in the literature. Following is a case series of five patients of diverse age groups who presented to the Emergency Medicine Department (ED) with a wide spectrum of clinical presentations, diverse clinical courses, and management strategies. These patients not only required the administration of anti-snake venom in the ED but also required additional interventional modalities including renal replacement therapy for acute kidney injury and biopsy-proven acute tubular necrosis, viper-induced consumptive coagulopathy, management of coagulopathy in pediatrics, management of compartment syndrome in pregnancy, mechanical ventilation for respiratory failure and management of neurotoxicity. We hereby emphasize that early recognition of signs of envenomation and early administration of anti-snake venom is imperative in the initial management of snakebites along with the monitoring of a snakebite victim for complications and timely management of the same.

## Introduction

According to the WHO, snake bite is a neglected public health issue in many tropical and subtropical countries. About 5.4 million snake bites occur each year, resulting in 1.8 to 2.7 million cases of envenoming. There are between 81,410 and 137,880 deaths and around three times as many amputations and other permanent disabilities each year. Snakebite envenoming is a neglected tropical disease that kills >100,000 people and maims >400,000 people every year [1]. Envenomation is the frequent manifestation, followed by coagulation abnormalities and neurotoxicity. Although snake bites are quite common in rural areas, urban centers do receive a decent number of snake bite cases mostly during damp and rainy seasons. Identifying the victims who require early administration of anti-snake venom is the key to the successful management of snake envenomation. India has long been thought to have more snakebites than any other country. However, inadequate hospital-based reporting has resulted in estimates of total annual snakebite mortality ranging widely from about 1,300 to 50,000 [2]. The following case series describes the WHO syndromic approach to snake bite and the tough task in the management of snake bite envenomation.

## Case presentation

Case 1

**Figure 1:**
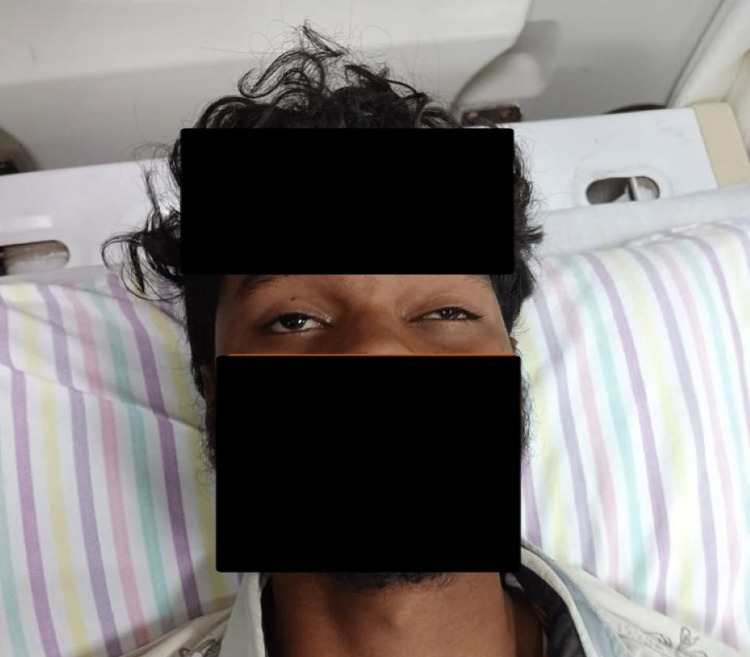
On presentation to the ED

A 36-year-old male presented to the ED with an alleged history of snake bite to the right foot 30 minutes prior to presentation. He complained of mild pain at the bite site with minimum bleeding. On the primary survey, he was found to be hemodynamically stable, and systemic examination was unremarkable. On local examination, there was swelling of the right foot up to the ankle with multiple bite marks with minimal bleeding. He was diagnosed to have local signs of envenomation and was administered 10 vials of anti-snake venom, and antibiotics and subsequently transferred to the intensive care unit. His initial laboratory parameters including Renal function tests, and coagulation parameters were within normal limits. The following day, the swelling progressed and I/V/O impending compartment syndrome, an emergency fasciotomy was done followed by the administration of 10 vials of Anti-snake venom. The patient’s renal functions gradually worsened on the fourth day of admission and was found to be anuric requiring six cycles of hemodialysis. However, the patient’s coagulation parameters worsened, anemia prevailed, and peripheral smear showed schistocytes, which aroused a strong suspicion of atypical hemolytic uremic syndrome hence four cycles of plasmapheresis were done for the same followed by five cycles of sustained low-efficiency dialysis (SLED). The patient’s general condition worsened with the development of pulmonary edema requiring the need for non-invasive ventilation. Despite the best resuscitative efforts patient however could not be revied and succumbed on the 14th day of snake bite. Post-mortem renal biopsy revealed features of severe acute tubular necrosis (Figure 1).

Case 2

**Figure 2:**
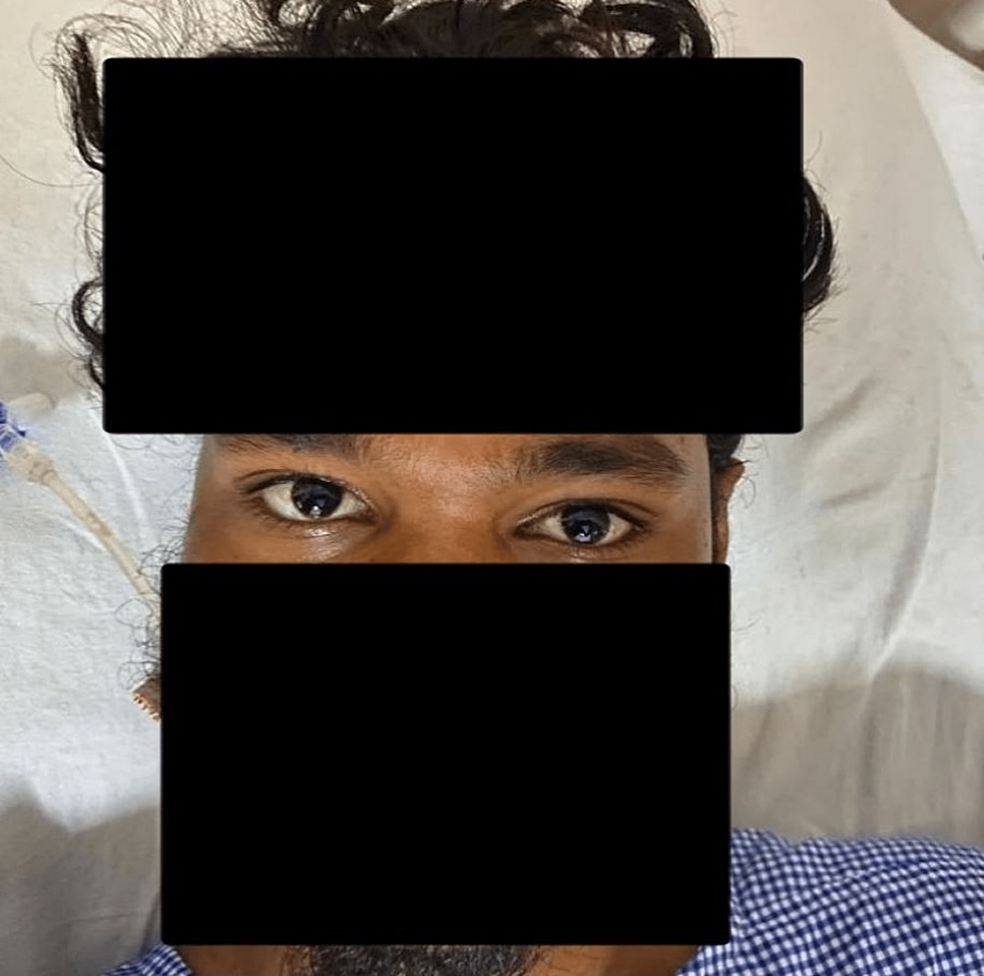
Patient on the day of discharge

A 24-year-old male was brought to the ED with a history of altered sensorium for 30 minutes. History revealed an alleged snake bite to the right foot two hours prior to the presentation. On the primary survey, the patient’s airway was compromised, and neck muscle wasting was noted with bilateral ptosis (Figure 1). The airway was secured by endotracheal intubation and mechanical ventilation was instituted. The patient was otherwise hemodynamically stable with other systemic examinations being unremarkable. There were no local signs of envenomation. Relevant Laboratory parameters revealed prothrombin time: 14.9 sec, INR: 1.34. The patient was hence diagnosed to have snake bite with neurological manifestation and coagulopathy. He was administered 10 vials of anti-snake venom followed by INJ ATROPINE 0.6MG IV prior to instituting two doses of INJ NEOSTIGMINE 0.04mg/kg IM one hour apart subsequently requiring an additional 10 vials of Anti-snake venom after six hours I/V/O persisting coagulopathy. The patient’s neurological status gradually improved and was extubated on day 2 of the presentation. The patient was discharged on day 3 of the presentation with neurological recovery (Figure 2).

Case 3

A 13-year-old boy was brought to the ED with an alleged history of snake bite to his right foot one hour prior to presentation. The patient complained of severe pain at the bite site. On further probing attenders give a history suggestive of the child having altered sensorium. On the primary survey, the child was found to have tachycardia, labored breathing, and a GCS of 9/15. The patient was intubated and mechanically ventilated for the same. Laboratory parameters revealed Prothrombin time: > 2 minutes and unrecordable INR. The patient was administered 10 vials of anti-snake venom, Vitamin K. After one hour, an additional 10 vials of anti-snake venom were administered I/V/O persisting coagulopathy, and Fresh Frozen plasma was administered. Non-contrast CT of the brain was however within normal limits. The patient’s condition gradually improved and was extubated on day 2 and was discharged on day 9 of the presentation.

Case 4

A 21-year-old G2P1L1 with 19 weeks of gestation was brought to the ED with an alleged history of snake bite to the right foot one hour prior to presentation. The patient complained of severe pain at the bite site. On the primary survey, she was hemodynamically stable. There was a gradual increase of swelling and laboratory parameters revealed thrombocytopenia. The patient was administered 10 vials of anti-snake venom followed by broad-spectrum antibiotics. The swelling gradually worsened with the development of cellulitis for which she underwent an emergency fasciotomy on day 2 of arrival. An additional 10 vials of anti-snake venom were administered I/V/O serial worsening of thrombocytopenia. Close fetal monitoring was done. The patient’s general condition improved with improvement in the platelet count and was subsequently discharged on day 10 of the presentation.

 Case 5

A 38-year-old male was brought to the EMD with an alleged history of snake bite three days back. The patient under the influence of alcohol accidentally held the snake in his right hand sustaining a bite injury over the left hand and while attempting to throw away the snake he sustained a bite injury over the right hand. The patient had developed progressive swelling of both the upper limbs. He was treated at a local ayurvedic center; however, he developed anuria for one day and was referred. On the primary survey, the patient was tachypneic, his systemic examination was otherwise unremarkable. Local examination revealed swelling of both the upper limbs up to the elbow without neurovascular compromise. His initial lab parameters revealed acute kidney injury and coagulopathy. He was administered 10 vials of anti-snake venom and hemodialysis was initiated. The following day he was noted to have a drop in hemoglobin with critical thrombocytopenia with persisting coagulopathy. He was diagnosed with viper-induced consumptive coagulopathy (VICC) and managed with blood and plasma transfusion, and additional doses of anti-snake venom. The patient’s general condition improved and was discharged on day 11 of presentation with hemodialysis at follow-up. However, on day 20 of the presentation, he returned to the ED with sudden onset breathlessness. History revealed non-compliance with hemodialysis. His clinical features were suggestive of flash pulmonary edema requiring emergency hemodialysis and was subsequently intubated on ventilator support. Despite the best resuscitative efforts, the patient succumbed on day 21 of the presentation.

## Discussion

The management of a snake bite victim is often challenging and intriguing. Snakebite is an important public health challenge. Venomous snake bites cause significant morbidity and mortality if treatment measures, especially antivenom therapy, are delayed. Thrombin-like enzymes (TLEs) are serine proteases, found mainly in pit viper venoms that induce coagulopathy. Hypofibrinogenemia is a usual result of fibrinogen degradation by TLEs [3]. Most of the viper-induced coagulopathy is attributed to this. The case series discussed above throws insight not only into the presentation of a venomous snake bite to the ED but also describes the different clinical courses the patient may undergo during their hospitalization. There are several cases reported on the initial clinical presentations in the literature, however, case series which include the presentation to ER, the hospital course, and management of complications have not been published in the literature.

We had five patients presenting with an alleged history of snake bites. Out of the five cases, three were males, one was primigravida, and one was in the pediatric age group. Four patients presented within one hour of the bite, and one had a delayed presentation. All of five cases had coagulopathy, out of which two cases developed disseminated intravascular coagulopathy. Three cases had local signs of envenomation and two cases had neurotoxicity. One was diagnosed to have VICC, and one patient had biopsy-proven renal tubular necrosis. Two patients underwent hemodialysis for acute kidney injury and two patients underwent fasciotomies for compartment syndrome. Two patients underwent intubation for respiratory failure, and two patients succumbed.

## Conclusions

Snake bite victims show varied clinical presentations ranging from local signs to systemic signs of envenomation. The patients who initially seem to be “stable” in ED, can have an erratic course and can land up in life-threatening complications, even death. Hence, it is of paramount importance for an emergency physician to recognize the signs of envenomation in ED, to administer anti-snake venom in time if envenomation exists, to have close monitoring of clinical and laboratory parameters to recognize if there are any additional doses of anti-snake venom required. It is important to timely secure the airway in cases of respiratory failure and profound shock, an eagle eye to recognize signs of compartment syndrome and involvement of a multi-disciplinary team for managing coagulopathy and acute kidney injury.

## References

[1] Gutiérrez JM, Calvete JJ, Habib AG, Harrison RA, Williams DJ, Warrell DA (2017). Snakebite envenoming. Nat Rev Dis Primers.

[2] Mohapatra B, Warrell DA, Suraweera W (2011). Snakebite mortality in India: a nationally representative mortality survey. PLoS Negl Trop Dis.

[3] Pradniwat P, Rojnuckarin P (2014). Snake venom thrombin-like enzymes. Toxin Rev.

